# Anlotinib reversed resistance to PD-1 inhibitors in recurrent and metastatic head and neck cancers: a real-world retrospective study

**DOI:** 10.1007/s00262-024-03784-5

**Published:** 2024-08-06

**Authors:** Jianyun Jiang, Bin Wu, Ying Sun, Jun Xiang, Chunying Shen, Xiayun He, Hongmei Ying, Zuguang Xia

**Affiliations:** 1 Department of Radiation Oncology, Fudan University Shanghai Cancer Center, Shanghai, 200032 China; 2 Department of Oncology, Shanghai Medical College, Fudan University, Shanghai, 200032 China; 3 Shanghai Clinical Research Center for Radiation Oncology, Shanghai, China; 4 Shanghai Key Laboratory of Radiation Oncology, Shanghai, 200032 China; 5 Department of Radiology, Fudan University Shanghai Cancer Centre, Shanghai, 200032 China; 6 School of Life Sciences and Biotechnology, Shanghai Jiao Tong University, Shanghai, 200240 China; 7 Department of Head and Neck Surgery, Fudan University Shanghai Cancer Center, Shanghai, 200032 China; 8 Department of Medical Oncology, Fudan University Shanghai Cancer Center, 270 Dong An Road, Shanghai, 200032 China

**Keywords:** Anlotinib, Programmed death-1 inhibitor, Resistance, Head and neck cancers

## Abstract

Patients with recurrent or metastatic head and neck cancers (R/M HNCs) are prone to developing resistance after immunotherapy. This retrospective real-world study aims to investigate whether the addition of anlotinib can reverse resistance to PD-1 inhibitors (PD-1i) and evaluate the efficacy and safety of this combination in R/M HNCs. Main outcomes included objective response rate (ORR), disease control rate (DCR), progression-free survival (PFS), overall survival (OS), duration of response (DOR), and safety. Potential biomarkers included PD-L1 expression, lipid index, and genomic profiling. Twenty-one patients with R/M HNCs were included, including 11 nasopharyngeal carcinoma (NPC), five head and neck squamous cell carcinoma (HNSCC), three salivary gland cancers (SGC), and two nasal cavity or paranasal sinus cancers (NC/PNC). Among all patients, ORR was 47.6% (95% CI: 28.6–66.7), with 2 (9.5%) complete response; DCR was 100%. At the median follow-up of 17.1 months, the median PFS and OS were 14.3 months (95% CI: 5.9-NR) and 16.7 months (95% CI:8.4-NR), respectively. The median DOR was 11.2 months (95% CI: 10.1-NR). As per different diseases, the ORR was 45.5% for NPC, 60.0% for HNSCC, 66.7% for SGC, and 50.0% for NC/PNC. Most treatment-related adverse events (TRAEs) were grade 1 or 2 (88.9%). The most common grades 3–4 TRAE was hypertension (28.6%), and two treatment-related deaths occurred due to bleeding. Therefore, adding anlotinib to the original PD-1i could reverse PD-1 blockade resistance, with a favorable response rate, prolonged survival, and acceptable toxicity, indicating the potential as a second-line and subsequent therapy choice in R/M HNCs.

**Supplementary Information:**

The online version contains supplementary material available at 10.1007/s00262-024-03784-5.

## Introduction

Head and neck cancers (HNCs) are among the most common malignancies worldwide [1]. Patients with relapsed or metastatic squamous cell carcinoma of the head and neck (R/M HNSCC) who progress after platinum-based therapy have a poor prognosis and no clear standard treatment. Median survival was approximately 1.8 months with optimal supportive care for platinum therapy-refractory HNSCC [2, 3]. Programmed cell death protein-1 checkpoint inhibitors (PD-1i) such as nivolumab and pembrolizumab showed promising clinical outcomes in relapsed or metastatic head and neck cancers (R/M HNCs); however, response rate was restricted to 15–20% [4–7]. The effectiveness of immune checkpoint inhibitors (ICIs) is limited by various mechanisms, including compensatory inhibitory pathways and the development of acquired resistance [8]. Alotinib is a new multi-target tyrosine kinase inhibitor [9] and has been shown in several preclinical studies to reverse resistance to immunotherapy by reshaping the immune microenvironment, including promoting tumor vascular normalization and inducing CD8^+^ T-cell infiltration into the tumor environment [10–12]. Clinically, several clinical studies demonstrated that combining anlotinib and PD-1 inhibitor conferred synergistic antitumor activity in refractory solid tumors [13, 14]. The latest clinical trial has demonstrated that the combination of camrelizumab and apatinib is highly effective in platinum-refractory, recurrent, or metastatic endemic NPC, with an impressive objective rate of 65.5% [15]. Previous trials across various cancer types have also reported positive outcomes by combining antiangiogenic and PD-1 inhibitors [16–19]. In this paper, we evaluated the synergistic clinical efficacy and safety of adding anlotinib to PD-1 inhibitors in advanced R/M HNCs. Additionally, we investigated the antitumor activity within key patient subgroups. Furthermore, through next-generation sequencing (NGS), we identified independent prognostic factors that have predictive value for patient survival.

## Materials and methods

### Study design and patients

This real-world retrospective study evaluated the efficacy and safety of anlotinib added to PD-1i therapy in R/M HNC patients who were resistant to PD-1i agents, including nasopharyngeal carcinoma (NPC), head and neck squamous cell carcinoma (HNSCC), salivary gland cancer (SGC), and nasal cavity or paranasal sinus cancers (NC/PNC). The collection of patient information has been approved by the Institutional Review Board (IRB) of Fudan University Shanghai Cancer Center (No. 1612167–18). The inclusion criteria were as follows: 1. Patients treated at our hospital between April 2021 and March 2023; 2. patients diagnosed with relapsed or metastatic head and neck cancers; and 3. patients who had developed resistance to PD-1i agents and were subsequently treated with anlotinib based on previous PD-1i agents. We utilized the electronic medical record system of our institute to retrospectively and consecutively identify patients. Ultimately, 21 eligible patients were included in our study. All 21 patients developed disease progression (PD) after receiving $\ge$1 systematic treatment. Number of previous systematic therapies before PD-1i plus anlotinib was recorded. Patients who had received prior treatment with PD-1 inhibitors continued to receive the same PD-1 inhibitors with the addition of anlotinib upon enrollment in our cohort.

### Treatment

Anlotinib was administered orally once daily (10 mg) for the first 2 weeks of a 21-day cycle. Intravenous administration of PD-1i, including pembrolizumab (200 mg), camrelizumab (200 mg), or sintilimab (200 mg), was given once every 21-day cycle in strict accordance with the prescribed drug instructions. If the daily dosage of anlotinib proved intolerable, it would be reduced to 8 mg per day. Treatment continued until PD, intolerable adverse effects, or mortality occurred. CT and MRI examinations were conducted before treatment and every two cycles during treatment, thereafter to evaluate changes in the target lesions as necessary until disease progression.

### Outcomes

The data cutoff was on August 19, 2023. Efficacy was assessed according to the RECIST version 1.1. The main evaluation indicators included objective response rate (ORR), disease control rate (DCR), progression-free survival (PFS), overall survival (OS), duration of response (DOR), time to response (TTR), and safety. ORR refers to the percentage of patients achieving complete response (CR) and partial response (PR). DCR refers to proportion of patients achieving an PR, CR, and stable disease (SD). PFS was calculated as the time from the 1st day of anlotinib administration to disease progression or death. OS was the duration from the 1st day of anlotinib administration to patient death or the last contact. DOR was calculated from the date of first response to progression or death. TTR was defined as the time to first response. Safety profile was closely monitored and assessed by experienced clinicians. Adverse events (AEs) were graded according to the CTCAE 4.02 and recorded throughout the study of administrating the combination of anlotinib and PD-1i.

### PD-L1 status

The tumor specimens, fixed in formalin and coated with paraffin, were stained with PD-L1 antibody (Clone 22 c3, Agilent) and evaluated by two pathologists to reach a consensus score. PD-L1 tumor proportion score (TPS) was defined as the percentage of tumor cells that are PD-L1 positive. Some specimens were deemed unevaluable for PD-1 expression due to insufficient tumor cell count or absence thereof. Combined positive score (CPS) was defined as the ratio of PD-L1-stained cells to the total number of viable tumor cells, multiplied by 100.

### Bioinformatics analysis of next-generation sequencing data

#### Sample extraction and sequencing (425 panels)

All samples were sequenced in a CLIA- and CAP-certified genomic testing facility (Nanjing Geneseeq Technology Inc., Nanjing, China). NGS was performed as described previously [20, 21], and *Supplementary Table 1* summarizes coverage and quality statistics for NGS sequencing in 18 patients. In brief, DNA from tumor samples and white blood cells were isolated using the QIAamp DNeasy Blood and Tissue kit (Qiagen, Dusseldorf, Germany) following the manufacturer’s instructions. Circulating cell-free DNA (cfDNA) was extracted using the QIAamp Circulating Nucleic Acid Kit (Qiagen, CA, USA). DNA fragments underwent end-repairing, A-tailing, and ligation with indexed adapters were selected as the size of 200 bp. Sequencing libraries were prepared by using the KAPA Hyper DNA Library Prep Kit (KAPA Biosystems, Wilmington, MA) according to the manufacturer’s protocol. Hybridization-based target enrichment was performed with customized xGen lockdown probes (Integrated DNA Technologies) targeting the exons and parts of introns of 425 cancer-relevant genes (Geneseeq prime®, Nanjing Geneseeq Technology Inc., Nanjing). Captured libraries were PCR-amplified with KAPA HiFi HotStart ReadyMix (KAPA Biosystems) followed by quantification using KAPA Library Quantification kit (KAPA Biosystems). DNA sequencing was performed on the HiSeq4000 NGS platform (Illumina) with a paired-end 150-bp read length.

#### Somatic and germline variant calling

After removing adapters and low-quality reads, reads were aligned to NCBI human genome reference assembly hg19 using the Burrows–Wheeler Aligner (BWA) algorithm, duplication sorting, realignment, and recalibration. During the mutation calling stage, the reads from the tumor sample were compared with the paired blood from the same patient to generate the somatic mutation list. The called somatic mutations were then filtered, meaning to retain only the mutations with the variant allele frequency (VAF) >  = 0.05 and supported by at least three reads, and annotated using the Variant Effect Predictor (VEP) package.

The GATK pipeline was used with default parameters to call the somatic and germline single-nucleotide variants (SNVs), including single-nucleotide polymorphisms (SNPs) and short insertion/deletions (INDELs). GATK standard pipeline was also used to do the somatic copy number variant (CNVs) discovery. During this stage, the reads from the tumor sample were compared with the paired blood from the same patient. If the paired blood was not available, a panel of normal (PON) samples was used instead for both CNVs and SNVs calling. The called somatic mutations were then filtered, retaining only the mutations with the VAF >  = 0.05 and supported by at least three reads, and annotated using the VEP package [22]. For the patients without paired blood, an additional filter VAF < 0.5 is applied when calling somatic mutations to filter out germline SNVs that may leak into the somatic results due to less matching of the PON and the tumor sample. We defined the copy number (CN) of a gene or segment 3–4 as gain, > 4 as amplification, 1–1.2 as loss, and < 1 as deletion, where CN 1.2–3 (around 2) is treated as normal. The mutations called from the blood samples were collected and filtered as the germline mutations.

#### TMB (tumor mutation burden) estimation

The TMB was defined as the total number of somatic SNP, and indel events detected per megabyte bases of tumor tissue[23]. As we conducted the NGS of the panel of 425 genes, we calculated the total exon size of all the genes in the panel, which is 2.235 Mb. The TMB was then estimated as the total number of somatic mutation events divided by 2.235.

### Prognosis and statistical analysis

Continuous variables were described using descriptive statistics, and categorical variables were presented as frequencies and percentages. The Kaplan–Meier method and log-rank test were used to analyze OS and PFS. The Python packages sksurv and sklearn were applied to explore the effect of certain mutations on survival of certain cohorts of patients. Log-rank test from lifelines package was performed to determine the significance between groups. All statistical analyses were completed using R or Python statistical software.

## Results

### Baseline characteristics

**Fig. 1:**
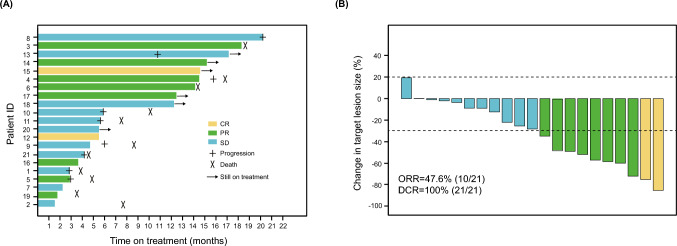
Efficacy analysis of primary endpoints. **A** Duration of responses of patients. The length of each bar represents the treatment duration for each patient. **B** Waterfall plot showing the best percentage change in target lesion size in patients with at least one postbaseline efficacy assessment (n = 21). DCR, disease control rate; ORR, objective response rate; PD, progressive disease; PR, partial response; and SD, stable disease

Twenty-one patients with R/M HNCs between April 2021 and March 2023 were included in our cohort. Their baseline characteristics were presented in detail **(**Table 1). Nineteen patients were males, and two were females. The median age was 53 years (range: 22–73). Of all, 11 patients were diagnosed with NPC, including five with HNSCC (two oropharynx squamous cell carcinoma, two laryngeal squamous cell carcinoma, and one hard plate squamous cell carcinoma), three with SGC (one parotid squamous carcinoma, one submandibular gland adenocarcinoma, not otherwise specified, and one submandibular gland carcinoma ex pleomorphic adenoma), and two with NC/PNC (one olfactory blastoma and one undifferentiated carcinoma of the nasal cavity). The majority (17/21) of pathological types were squamous cell carcinomas (11 NPC, five HNSCC, and one SGC). The majority of patients have undergone more than three lines of prior treatment. Sixteen patients developed acquired resistance to PD-1i, and the remaining five had primary resistance. The types of PD-1i the patients previously received included sintilimab (15/21), camrelizumab (4/21), and pembrolizumab (2/21). The relapse sites were mainly head and neck (11/21), followed by lung (9/21), bone (5/21), and liver (4/21). None of our patients experienced brain metastases. PD-L1 expression CPS was available for 19 patients of whom 14 were found to be positive (CPS $\ge$ 1). The median follow-up time in our patient cohort was 17.1 months (range: 3.4–26.2; data cutoff: August 19, 2023). The median duration of previous anti-PD1 treatment was 7.7 months (range: 1.5–17.7), and the median duration of the combination treatment was 15.2 months (range: 1.5–20.2). As of the data cutoff time, six of 21 patients continued with ongoing treatment (Fig. 1A). The remaining 15 patients discontinued the medication, including 11 due to disease progression, three due to adverse effects, and one patient stopped taking the treatment based on personal choice.

**Table 1:** Patient characteristics

Characteristic	Patients (n = 21)
Sex, n (%)	
Male	19 (90.5%)
Female	2 (9.5%)
Age (years), median (range)	53 (22–73)
Tumor type	
NPC	11 (52.4%)
HNSCC^*^	5 (23.8%)
SGC^#^	3 (14.3%)
NC/PNC^†^	2 (9.5%)
Pathology	
Squamous	17 (81.0%)
Non-squamous	4 (19.0%)
Current treatment lines	
2	1 (4.8%)
3	6 (28.6%)
4	5 (23.8%)
5	5 (23.8%)
6	3 (14.3%)
8	1 (4.8%)
Type of anti-PD-1 resistance	
Innate	5 (23.8%)
Acquired	16 (76.2%)
Anti-PD-1 drugs	
Sintilimab	15 (71.4%)
Camrelizumab	4 (19.0%)
Pembrolizumab	2 (9.5%)
Site of relapse	
Head and neck	11 (52.4%)
lung	9 (38.1%)
Bone	5 (23.8%)
Liver	4 (19.0%)
TPS of PD-L1	
< 1	10 (52.6%)
1–49	6 (31.6%)
≥ 50	3 (15.8%)
CPS of PD-L1	
< 1	5 (26.3%)
1–9	7 (36.8%)
≥ 10	7 (36.8%)
MSI	
Non-MSI-H	15 (71.4%)
MSI-H	1 (4.8%)
MS-S	1 (4.8%)
Unknown	4 (19.0%)
TMB	
≥ 10 Muts/Mb	1 (4.8%)
< 10 Muts/Mb	13 (61.9%)
0 Muts/Mb	4 (19.0%)
Unknown	3 (14.3%)

*NPC* nasopharyngeal carcinoma, *HNSCC* head and neck squamous cell carcinoma, *SGC* salivary gland cancers, *NC/PNC* nasal cavity or paranasal sinus cancers, *TPS* tumor proportion score, *CPS* combined positive score, *MSI* microsatellite instability, and *TMB* tumor mutation burden

^*^HNSCC included two oropharynx squamous cell carcinoma, two laryngeal squamous cell carcinoma, and one hard plate squamous cell carcinoma

^#^SGC included one parotid squamous carcinoma, one submandibular gland adenocarcinoma, not otherwise specified, and one submandibular gland carcinoma ex pleomorphic adenoma

^†^NC/PNC included one olfactory blastoma and one undifferentiated carcinoma of the nasal cavity

### Efficacy evaluation

All patients were given anlotinib in combination with their PD-1i agents. The overall response evaluation of the cohort is summarized in *Supplementary Table 2.* Two achieved complete response (CR), eight achieved partial response (PR), and eleven achieved stable disease (SD). The ORR was 47.6% (95% CI: 28.6–66.7%), and the DCR was 100.0%. Figure 1B shows changes in target lesions from baseline during treatment. The median TTR (mTTR) was 3.47 months (95% CI: 2.37-NA). Furthermore, the response to treatment was long-lasting, with a median DOR (mDOR) of 11.2 months (95% CI: 10.1-NA) (*Supplementary Fig. 1)*. Notably, four patients had a maintenance period exceeding 10 months. Moreover, we also evaluated the objective responses of different resistance types and CPS/TPS subgroups, as shown in *Supplementary Tables 3* and *4*, respectively.

**Fig. 2:**
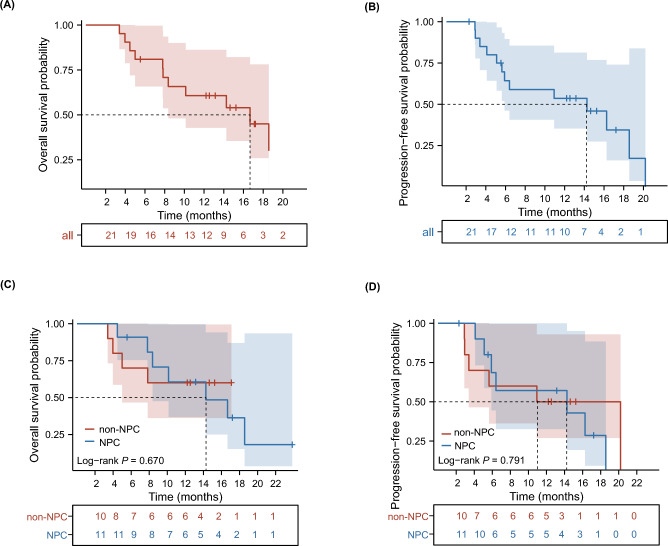
Kaplan–Meier curves were performed to assess OS (**A**, **C**) and PFS (**B**, **D**) of patients. OS, overall survival and PFS, progression-free survival

Up to the date of data cutoff, 13 patients (61.9%) had progressed, and 11 patients (52.4%) had died. The median OS (mOS) was 16.7 months (95% CI: 8.4-NR), and the 12-month OS rate was 61.2% (95%CI: 42.1–88.9%) (Fig. 2A). The median PFS (mPFS) was 14.3 months (95% CI: 5.9-NR), and the 12-month PFS rate was 58.9% (95% CI: 37.7–92.2%) (Fig. 2B). The patients with NPC exhibited a favorable mOS of 14.3 months (95% CI: 8.4-NR) and mPFS of 14.3 months [(95% CI: 5.9-NR) (Fig. 2C and 2D*,* respectively)]. Survival analysis of different patient subgroups is summarized in *Supplementary Table 2*.

### Safety evaluation

The treatment-related adverse events (TRAEs) are presented in Table 2. Most TRAEs were grade 1 or 2 (88.9%). The most frequent grade 1 or 2 AEs were loss of appetite (14/21), fatigue (13/21), low sodium (12/21), weight loss (11/21), and mucositis oral (10/21). The most frequent grade 3 TRAE observed were hypertension (6/21), followed by fatigue (2/21), low sodium (2/21), and pharyngolaryngeal pain (2/21). Anlotinib dosage was adjusted from 10 to 8 mg/day in six individuals, while the PD-1i dosage remained unchanged in all patients. Three patients discontinued medication due to intolerance (specifically, severe nose bleeding, parapharyngeal infection, and widespread exfoliative dermatitis), and two patients died from excessive bleeding.

**Table 2:** Treatment-related adverse events

Events	Grades 1–2	Grade 3	Grade 4	Grade 5
Neutrophil reduction	1	0	0	0
Anemia	5	0	0	0
Thrombocytopenia	1	0	0	0
Elevated TBIL	3	0	0	0
Elevated ALT	3	0	0	0
Elevated AST	6	0	0	0
Elevated γ-GT	4	1	0	0
Elevated creatinine	2	0	0	0
Low sodium	12	2	0	0
Low potassium	0	1	0	0
Elevated T-CHO	4	1	0	0
Elevated TG	9	0	0	0
Hypothyroidism	8	0	0	0
Proteinuria	6	0	0	0
Urinary tract infection	1	0	0	0
Upper respiratory infection	3	0	0	0
Hypertension	2	6	0	0
Hypotension	3	0	0	0
Hand–foot syndrome	9	0	0	0
Rash or pruritus	3	1	0	0
Fatigue	13	2	0	0
Loss of appetite	14	0	0	0
Headache	5	1	0	0
Diarrhea	9	1	0	0
Nausea or vomiting	2	0	0	0
Dysphagia	1	1	0	0
Pharyngolaryngeal pain	5	2	0	0
Mucositis oral	10	1	0	0
Gum pain	1	0	0	0
Hemorrhage	8	0	0	2
Nasopharyngeal/nasal bleeding	2	0	0	1
Throat bleeding	1	0	0	0
Neck bleeding	0	0	0	1
Gastrointestinal bleeding	1	0	0	0
Urinary occult blood	1	0	0	0
Fecal occult blood positive	4	0	0	0
Hoarseness	6	1	0	0
Cough	5	0	0	0
Arthralgia	4	0	0	0
Pneumonitis	4	0	0	0
Dysgeusia	1	0	0	0
Insomnia	1	0	0	0
Weight loss	11	0	0	0

*TBIL* total bilirubin, *AST* aspartate aminotransferase, *ALT* alanine aminotransferase, *γ-GT, γ*-glutamyl transpeptidase, *T-CHO* total cholesterol, and *TG* triglyceride

### Biomarker analysis

In our cohort, the PD-L1 CPS $\ge$ 10 group exhibited an extended PFS and OS compared to the CPS $<$ 10 group (PFS: HR = 0.339 [95% CI: 0.104–1.107] and OS: HR = 0.270 [95% CI: 0.073–0.998]), but the difference was also not statistically significant (PFS: *P* = 0.053 and OS: *P* = 0.058) (*Supplementary Fig. 2A and 2B,* respectively).

**Fig. 3:**
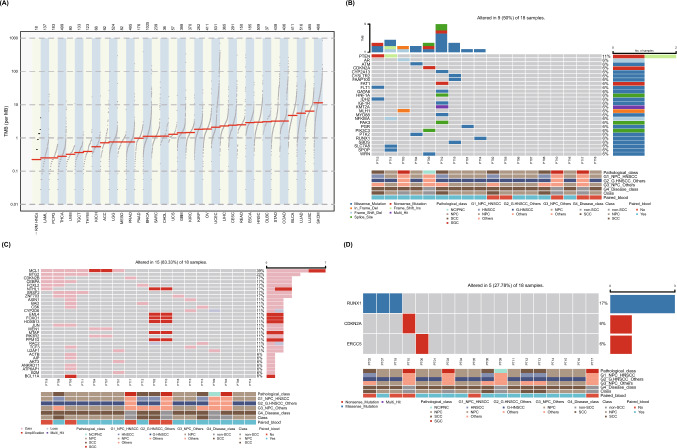
**A** The TMB of the R/M HNCs cohort was compared with that of all TCGA cancers. **B** All somatic mutated genes were plotted. **C** Significant germline mutations were identified and analyzed. **D** Three germline mutations (RUNX1, CDKN2A, and ERCC5) which could cause effects on cancer development were detected. TMB, tumor mutational burden. NPC, nasopharyngeal cancer, SCC, squamous cell carcinoma; HNSCC, head and neck squamous cell carcinoma; HNSCCg, generalized HNSCC; SGC, salivary gland cancer; and NC/PNC, nasal cavity or paranasal sinus cancer

We estimated the TMB of our R/M HNCs cohort, and our cohort showed relatively low TMB value in across all the TCGA cancers (Fig. 3A). The cohort consists of NPC (nasopharyngeal cancer), SCC/HNSCC (squamous cell carcinoma/head and neck squamous cell carcinoma), SGC (salivary gland cancer), and NC/PNC (nasal cavity or paranasal sinus cancer). All somatic mutated genes are plotted in Fig. 3B, mutation frequency of PTEN is 2, and all other genes such as CDKN2A, ATM, FAT1, and IDH2 only occur once. There are nine patients with no somatic SNVs detected. We analyzed somatic copy number variant (CNV), most of the CNV events are gain and amplification, the MCL1 (39%) and BTG2 (22%) donate the most frequently copy number increased genes in the cohort (Fig. 3C). We checked the significant germline mutations, the mutations which could cause effects on cancer development or progression, only very three gene mutations were detected, including RUNX1, CDKN2A, and ERCC5 (Fig. 3D). We found that there are very less somatic genomic change events in the R/M HNCs.

Further, we want to find whether there is any difference in the genomic mutations between these different cancers, so we divided the cohort into several comparison groups: G1, NPC vs HNSCC, G2, generalized HNSCC (HNSCCg) vs the others (non-HNSCCg), G3, NPC vs the others (non-NPC), and G4, SCC vs non-SCC (for detail patients enrolled, see *Supplementary Table 5*). As the somatic mutations are quite few, so we did not detect any differences in between the four comparison groups. There are also no notable disparities observed in OS or PFS between G1 ~ G4 (*Supplementary Figs. 3 and 4,* respectively).

**Fig. 4:**
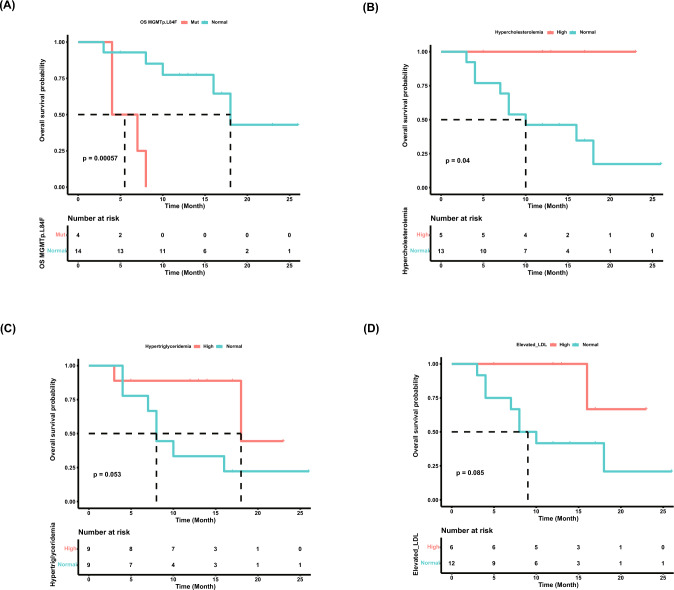
The identification of biomarkers with prognostic value. **A** The effect of MGMT L84F SNP on OS. **B** The effect of cholesterol level on OS. **C** The effect of triglyceride level on OS. **D** The effect of LDL level on OS. OS, overall survival

The low TMB in the cohort limits the identification of biomarkers between somatic mutations and prognosis. We want to find another molecular index to accomplish this. Germline SNV is a germline substitution of a single nucleotide or a small insertion or deletion of several bases at a specific position in the genome that is present in a sufficiently large fraction of considered population [24, 25] which can be detected by control blood sample sequencing. We identified 3598 germline SNVs (*Supplementary Table 6*), making it easy to find biomarkers within these cancer types. We focused on SNP events which cause amino acid changes and carried out LASSO regression to find out the most relevant SNP as the biomarker, depending on which the patients could be divided into SNP carrier and non-carrier groups with the median OS of the two groups showing significant difference in the whole cohort and NPC patients, for the NPC patients being the homogeneous group with the most patients **(**Fig. 3B*, Pathological class*). To eliminate the effect of incidental events on our results, only the SNVs which had occurred in more than 3 (≥ 4) patients were preserved. A list of 15 SNPs of 14 genes was found (*Supplementary Table 7*), among these, the SNP (dbSNP ID rs12917) which causes the protein change L84F of MGMT (O-methylguanine-DNA-methyltransferase) is the most significant biomarker with adjusted p values < 0.05 which behaves like a hazard factor to the prognosis of the patients. The SNP carriers showed a median OS of 5.5 months, and SNP-free patients were 18 months with significant log-rank test *P* = 0.00057 (Fig. 4A). This SNP is reported to be a risk factor on prognosis of many cancers [26–30], we hypothesize this to be a potential SNP which contributes to the poor prognosis of the drug treatment. We also confirm the effect of MGMT L84F SNP on PFS (*P* = 0.00035) of the cohort (*Supplementary Fig. 5A*).

We also analyzed the state of hypercholesterolemia, hypertriglyceridemia, and LDL elevation of the patients at the end point of drug treatment and found that high levels of the lipid in blood showed a strong correlation with good OS (Fig. 4B–D) and PFS (*Supplementary Fig. 5B–D*). Among these lipid indexes, hypercholesterolemia showed the strongest effect on OS (*P* = 0.04), with all the patients with hypercholesterolemia do not decrease at the end of the follow-up.

## Discussion

We presented the first real-world retrospective study that adding anlotinib to PD-1 inhibitors to treat refractory HNCs that developed resistance to PD-1 inhibitors. In our study, all patients received the same PD-1i before and after progression, which validates the contribution of anlotinib monotherapy in reversing resistance. Our results yielded a favorable ORR of 47.6% and DCR of 100.0%, despite the fact that over two-thirds of patients received three-line or more treatment regimens. Many clinical trials have shown promising results with the initial use of both drugs. Our study presents novel evidence indicating that adding anlotinib to PD-1 inhibitors is highly effective, which supports the idea that sequential treatment with anlotinib after PD-1i resistance can also be clinically beneficial without increasing premature discontinuation due to adverse effects of the two drugs.

With the increasing use of immunotherapy in clinical settings, acquired resistance has emerged as a significant obstacle. The resistance mechanism to immunotherapy is complicated, with many components in TME promoting immune evasion [8]. VEGF-mediated immunosuppression plays a crucial role in inhibiting DC maturation, reducing T-cell infiltration into tumors, and promoting inhibitory signaling [31, 32]. It has been reported that combining antiangiogenic agents with PD-1 inhibitors can have a synergistic antitumor effect [33, 34]. For instance, clinical trials using nivolumab plus regorafenib and pembrolizumab plus lenvatinib have confirmed the effectiveness of this regimen in later-line treatments [17–19, 35]. Similarly, a recent clinical trial combining apatinib and camrelizumab in R/M NPC patients showed encouraging efficacy, with an ORR of 65.5%, a DCR of 86.2%, and a median PFS of 10.4 months [15]. Anlotinib has been proven effective against various cancers [36–38]. Anlotinib monotherapy showed promising results in a phase II study (NCT03906058) for R/M NPC patients as a palliative treatment, achieving a DCR of 77.8%, ORR of 22.2%, mPFS of 5.7 months, and mOS of 23.9 months. Their study comprised half of patients whose previous PD-1 inhibitor therapy had failed, indicating that anlotinib may represent an option following immunotherapy failure. In our study, 11 patients with NPC achieved a DCR of 100.0% and an ORR of 45.5%. The mPFS and mOS were 14.3 months (95% CI: 5.9-NR) and 14.3 months (95% CI: 8.4-NR), respectively. Our results suggested that the combination of anlotinib and PD-1i may represent a more effective strategy for prolonging PFS than anlotinib monotherapy in cases where PD-1i treatment has failed or resistance has emerged. Various combination therapies involving anlotinib have been clinically tested for NPC treatment both as a first-line and later-line option. Clinical trials such as NCT04736810, NCT05198531, and NCT04996758 are currently underway.

Regarding R/M HNSCC, the phase II study of penpulimab plus anlotinib for R/M HNSCC after first-line chemotherapy failure showed an ORR of 28.0% and DCR of 84.0% [39]. In a phase II clinical trial (NCT04999800), the combination of anlotinib and pembrolizumab as a first-line treatment achieved an ORR of 46.7% and a DCR of 100% in the CPS ≥ 1 subgroup. Our result showed a 60.0% ORR and 100.0% DCR in R/M HNSCC patients who had undergone multiple lines of treatment, moderately superior to the results reported in these trials. As for R/M SGC, a phase II study evaluating anlotinib monotherapy showed a DCR of 81.0% and an ORR of 19.1% [40]. In our study of three patients with RMSGC, one patient achieved CR, another PR, and the third had SD, but a larger patient cohort is needed to validate this result. A published phase II trial demonstrated promising efficacy and safety of combining anlotinib with PD-1i in treating refractory solid tumors, with an ORR of 22.0% and a DCR of 73.2% [14]. Taken together, the combined use of anlotinib and PD-1i has been increasingly investigated for their synergistic effects in various types of cancer. Interestingly, one patient with oropharyngeal cancer in our cohort developed resistance to both PD-1i and anlotinib monotherapy; however, the patient achieved PR and did not experience disease progression with a PFS of over 12.5 months. This finding supports evidence for the synergistic mechanism between antotinib and PD-1 inhibitors in overcoming mutual resistance [10, 13, 41, 42].

The safety profile of adding anlotinib to PD-1 inhibitors was consistent with the previous trials of anlotinib and PD-1 inhibitor monotherapy trials [13, 43–45]. The occurrence of unexpected adverse events was not observed, and any side effects were acceptable through supportive care medications, treatment interruption, or discontinuation. Hypertension was a common grade 3 or higher adverse effect and can be effectively managed [13, 17, 46]. The previous research has indicated that the administration of anti-VEGF(R) therapies may be associated with an elevated susceptibility to bleeding [47, 48], and there were two treatment-related deaths due to bleeding. TRAEs result in dose reductions and interruption for 6 (28.6%) and 3 (14.3%) patients, respectively. The median time to dose reduction was approximately 3.3 months (95% CI: 2.5 months-NR).

We have found that the SNP rs12917 MGMT L84F is a hazard marker of the anlotinib and PD-1i treatment. Patients with this SNP showed a very poor prognosis. It may serve as a potential biomarker for stratifying patients who demonstrate resistance to PD-1i and might benefit from the addition of anlotinib. We also observed that hypercholesterolemia, hypertriglyceridemia, and LDL elevation are correlative to the good prognosis of the treatment. This observation indicates that blood lipid level, especially cholesterol level, may be predictive to response to anlotinib and PD-1i treatment or may interact with the anlotinib and PD-1i treatment resulting in a good prognosis. More studies are needed to explain the role of high cholesterol levels with the good prognosis, the anlotinib treatment, and PD-1i treatment, including which one is a trigger to or the result of the others.

Our study encountered several limitations. Firstly, the heterogeneity of head and neck cancer varies based on the specific locations of tumors and treatment approaches, which might influence the identification of significant prognostic factors. Secondly, there is variability in the use of PD-1 inhibitors among patients. Participants were required to continue their existing PD-1 inhibitor, resulting in the inclusion of three distinct PD-1 inhibitors within this research. Although no statistical differences in efficacy were observed among these three inhibitors, potential bias may exist. In addition, given the small sample size, caution should be exercised when interpreting genomic profiling results and larger cohorts are needed for future analysis. In conclusion, the favorable efficacy of anlotinib added to PD-1 inhibitor therapy in our study supports the exploration of PD-1 inhibitors plus multi-target TKI treatment strategy in the immune-resistant population. Our comprehensive genomic profiling can help identify predictive biomarkers for refractory head and neck cancers treated with this combination therapy. Moreover, our results also prompt consideration of whether it would be more advantageous to prioritize a combination therapy involving PD-1i and antiangiogenic TKIs or PD-1i monotherapy, followed by subsequent treatment with antiangiogenic TKIs to mitigate side effects, for patients with refractory head and neck cancers.

## Supplementary Information

Below is the link to the electronic supplementary material.

Supplementary file1 (DOCX 25 kb)

Supplementary file2 (PDF 95 kb)

Supplementary file3 (PDF 476 kb)

Supplementary file4 (PDF 403 kb)

Supplementary file5 (PDF 400 kb)

Supplementary file6 (PDF 425 kb)

Supplementary file7 (XLSX 18 kb)

Supplementary file8 (XLSX 16 kb)

Supplementary file9 (XLSX 2755 kb)

Supplementary file10 (XLSX 68 kb)

Supplementary file11 (DOCX 13 kb)

## Data Availability

The data supporting the findings of this study can be obtained upon request from the corresponding author.

## Abbreviations

AEs: Adverse events
AST: Aspartate aminotransferase
ALT: Alanine aminotransferase
BWA: Burrows–Wheeler aligner
CN: Copy number
CNVs: Copy number variant
CPS: Combined positive score
CR: Complete response
DCR: Disease control rate
DOR: Duration of response
γ-GT: γ- Glutamyl transpeptidase
HNSCC: Head and neck squamous cell carcinoma
ICIs: Immune checkpoint inhibitors
LASSO: Least absolute shrinkage and selection operator
MGMT: O-methylguanine-DNA-methyltransferase
NC/PNC: Nasal cavity or paranasal sinus cancer
NGS: Next-generation sequencing
NPC: Nasopharyngeal carcinoma
ORR: Objective response rate
OS: Overall survival
PD: Disease progression
PD-1i: PD-1 inhibitors
PFS: Progression-free survival
PR: Partial response
R/M HNCs: Recurrent or metastatic head and neck cancers
SCC: Squamous cell carcinoma
SD: Stable disease
SGC: Salivary gland cancer
SNPs: Single-nucleotide polymorphisms
SNVs: Single-nucleotide variants
TMB: Tumor mutation burden
TPS: Tumor proportion score
TRAEs: Treatment-related adverse events
TTR: Time to response
TBIL: Total bilirubin
T-CHO: Total cholesterol
TG: Triglyceride
VAF: Variant allele frequency
VEGF: Vascular endothelial growth factor
VEP: Variant effect predictor
