# Supplementary material for: Anlotinib reversed resistance to PD-1 inhibitors in recurrent and metastatic head and neck cancers: a real-world retrospective study
Source: Cancer Immunol Immunother. 2024 Aug 6;73(10):199. doi: 10.1007/s00262-024-03784-5 (PMC11303650; doi:10.1007/s00262-024-03784-5)
Supplement: Supplementary file 1 — Supplementary file1 (DOCX 25 kb) [file 262_2024_3784_MOESM1_ESM.docx]

**Supplementary table 2** Tumor response and survival outcomes

| Outcomes | Total (n=21) | NPC (n=11) | HNSCCs (n=5) | SGC (n=3) | NC/PNC (n=2) |
| --- | --- | --- | --- | --- | --- |
| ORR (n, %) | 10 (47.6) | 5 (45.5) | 3 (60.0) | 2(66.7) | 1 (50.0) |
| DCR (n, %) | 21 (100.0) | 11 (100.0) | 5 (100.0) | 3 (100.0) | 2 (100.0) |
| CR (n, %) | 2 (9.5) | 1 (9.1) | 0 (0.0) | 1 (33.3) | 0 (0.0) |
| PR (n, %) | 8 (38.1) | 4 (36.4) | 3 (60.0) | 1 (33.3) | 1 (50.0) |
| SD (n, %) | 11 (52.4) | 6 (54.5) | 2 (40.0) | 1 (33.3) | 1 (50.0) |
| PD (n, %) | 0 (0.0) | 0 (0.0) | 0 (0.0) | 0 (0.0) | 0 (0.0) |
| Median PFS (months) | 14.3 (5.9-NR) | 14.3 (5.9-NR) | 5.6 (3.4-NR) | NE^*^ | NE |
| 12-month PFS (%) | 58.9 (37.7-92.2) | 57.1 (32.6-100.0) | 40 (13.7-100.0) | NE | NE |
| Median OS (months) | 16.7 (8.4-NR) | 14.3 (8.4-NR) | 7.8 (5.0-NR) | NE | NE |
| 12-month OS (%) | 61.2 (42.1-88.9) | 60.6 (36.8-99.8) | 40 (13.7-100.0) | NE | NE |

Abbreviation: NPC: nasopharyngeal carcinoma; HNSCC: head and neck squamous cell carcinoma; SGC: salivary gland cancers; NC/PNC: nasal cavity or paranasal sinus cancers; ORR: objective response rate; DCR: disease control rate; CR: complete response; PR: partial response; SD: stable disease; PD: disease progression; PFS: progression-free survival; OS: overall survival; NR, not reached; NE: not evaluable.

^*^ Survival for patients with SGC and NC/PNC could not be assessed due to the limited events.

**Supplementary Table 3** Types of PD-1 inhibitor resistance and treatment response

| Resistance to PD-1i agent | | Best overall response | ORR; DCR |
| --- | --- | --- | --- |
| Innate (n=5) |  | PR:3; SD:2; | 60.0%; 100.0% |
| Acquired (n=16) |  | PR:7; SD: 8; PD:1 | 43.8%; 93.8% |

Abbreviation: ORR: objective response rate; DCR: disease control rate; PR: partial response; SD: stable disease; PD: disease progression

**Supplementary Table 4** PD-L1 status and treatment response

|  | CR | PR | SD | ORR |
| --- | --- | --- | --- | --- |
| Known CPS (n=19) |  |  |  |  |
| <1 (n=5) | 0 | 1 | 4 | 20.0% |
| ≥1 (n=14) | 2 | 5 | 7 | 50.0% |
| Known TPS (n=19) |  |  |  |  |
| <1 (n=10) | 2 | 2 | 6 | 40.0% |
| ≥1 (n=9) | 0 | 4 | 5 | 44.4% |

Abbreviation: CPS: combined positive score; TPS: tumor proportion score; CR: complete response; PR: partial response; SD: stable disease; ORR: objective response rate
