# Supplementary figures and images for: Anlotinib reversed resistance to PD-1 inhibitors in recurrent and metastatic head and neck cancers: a real-world retrospective study
Source: Cancer Immunol Immunother. 2024 Aug 6;73(10):199. doi: 10.1007/s00262-024-03784-5 (PMC11303650; doi:10.1007/s00262-024-03784-5)

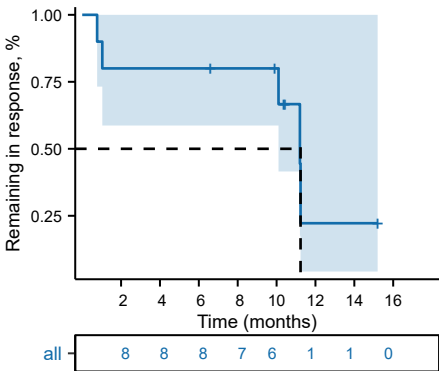

Supplement: Supplementary file 2 — Supplementary file2 (PDF 95 kb) [file 262_2024_3784_MOESM2_ESM.pdf]

(A)

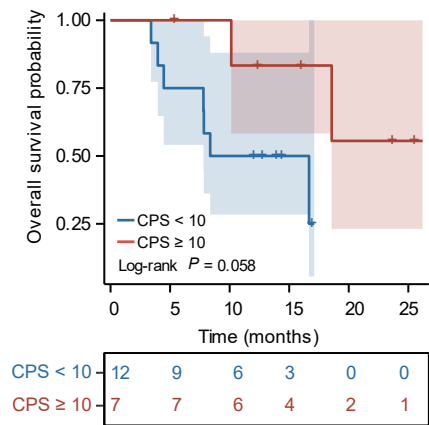

(B)

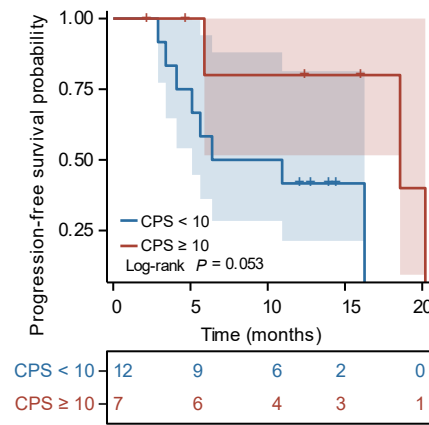

Supplement: Supplementary file 3 — Supplementary file3 (PDF 476 kb) [file 262_2024_3784_MOESM3_ESM.pdf]

(A)

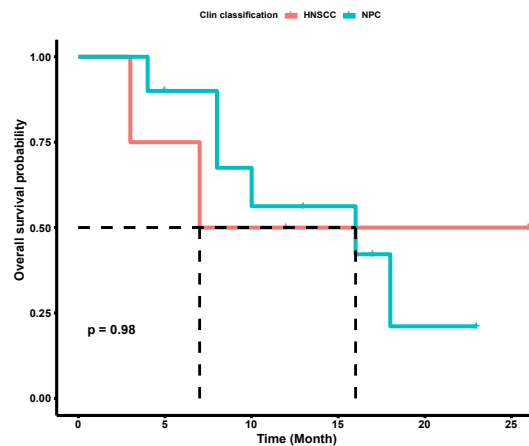

(B)

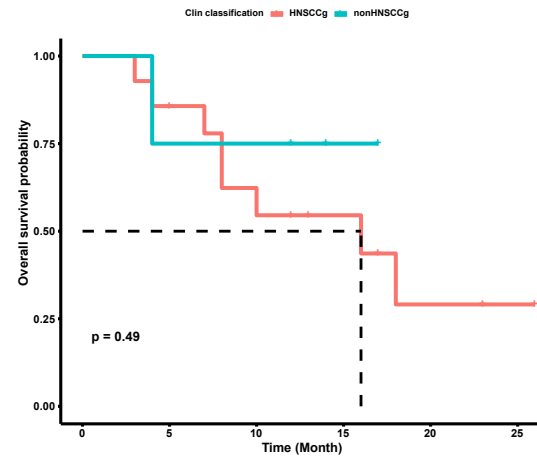

(C)

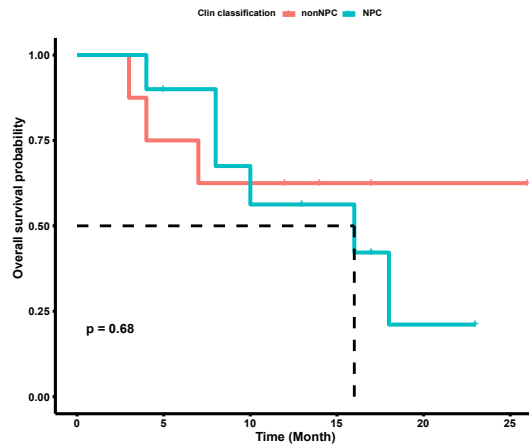

(D)

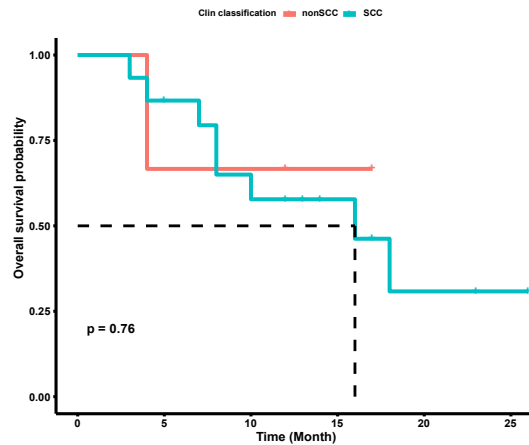

Supplement: Supplementary file 4 — Supplementary file4 (PDF 403 kb) [file 262_2024_3784_MOESM4_ESM.pdf]

(A)

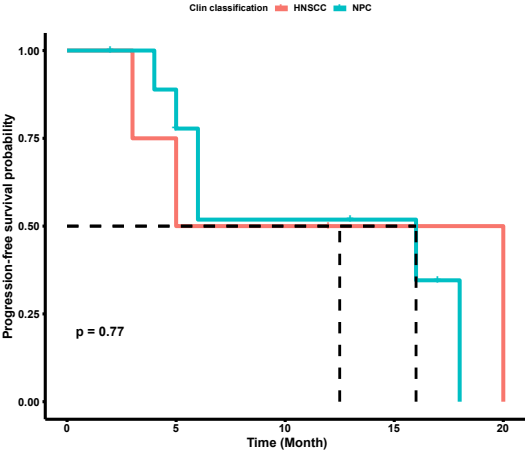

(B)

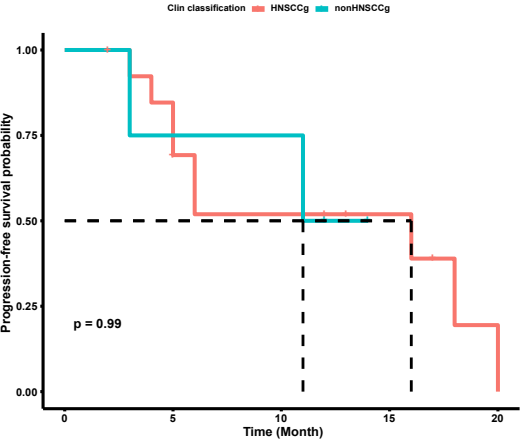

(C)

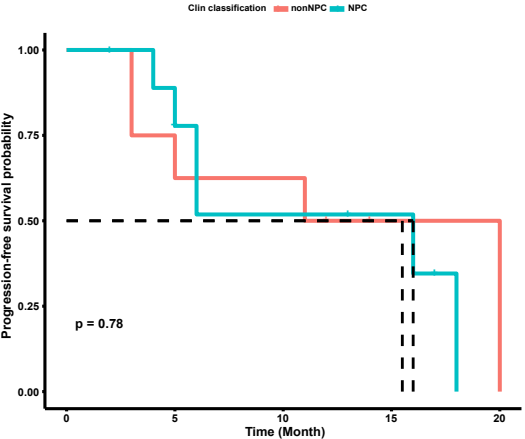

(D)

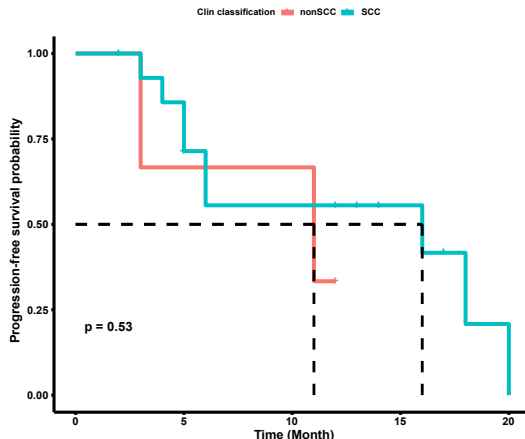

Supplement: Supplementary file 5 — Supplementary file5 (PDF 400 kb) [file 262_2024_3784_MOESM5_ESM.pdf]

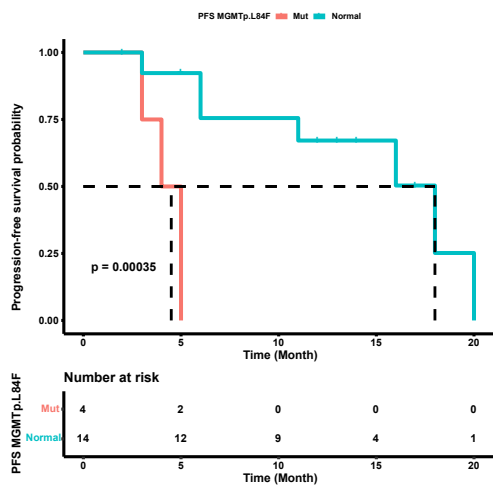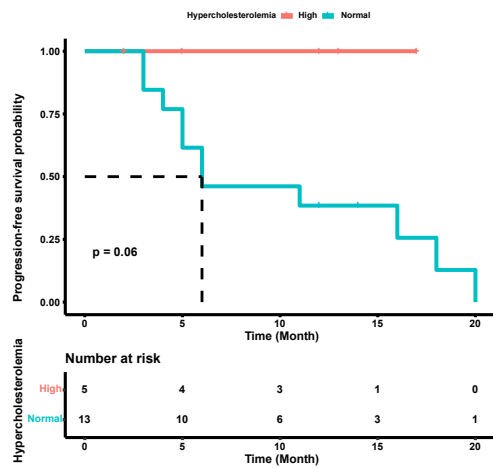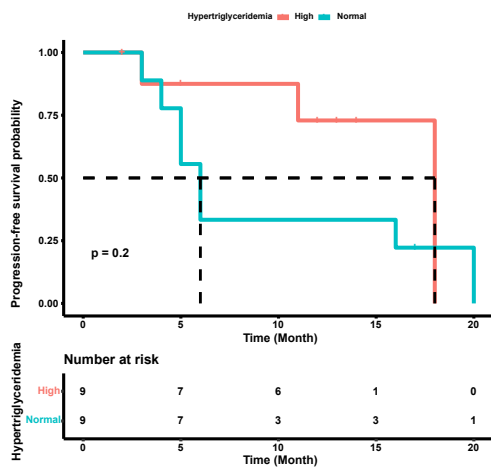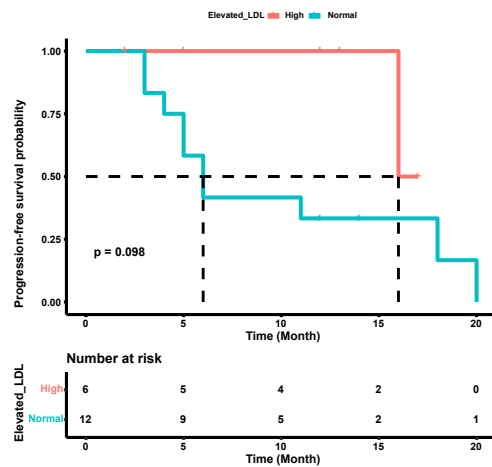

Supplement: Supplementary file 6 — Supplementary file6 (PDF 425 kb) [file 262_2024_3784_MOESM6_ESM.pdf]
