# Supplementary material for: Anlotinib reversed resistance to PD-1 inhibitors in recurrent and metastatic head and neck cancers: a real-world retrospective study
Source: Cancer Immunol Immunother. 2024 Aug 6;73(10):199. doi: 10.1007/s00262-024-03784-5 (PMC11303650; doi:10.1007/s00262-024-03784-5)
Supplement: Supplementary file 11 — Supplementary file11 (DOCX 13 kb) [file 262_2024_3784_MOESM11_ESM.docx]

**Supplementary Figure legends**

**Supplementary Figure 1:** Kaplan–Meier curve showing the duration of response.

**Supplementary Figure 2:** Kaplan–Meier curves comparing the OS (A) and PFS (B) between patients with PD-L1 CPS $\geq$10 and those with CPS$<$10. OS, overall survival; PFS, progression-free survival; CPS: combined positive score.

**Supplementary Figure 3:** Kaplan–Meier curves showing OS between G1~G4 groups. OS, overall survival; NPC, nasopharyngeal cancer, SCC, squamous cell carcinoma; HNSCC, head and neck squamous cell carcinoma; HNSCCg, generalized HNSCC.

**Supplementary Figure 4:** Kaplan–Meier curves showing PFS between G1~G4 groups. PFS, progression-free survival; NPC, nasopharyngeal cancer, SCC, squamous cell carcinoma; HNSCC, head and neck squamous cell carcinoma; HNSCCg, generalized HNSCC.

**Supplementary Figure 5:** The identification of biomarkers with prognostic value. (A) The effect of MGMT L84F SNP on PFS. (B) The effect of cholesterol level on PFS. (C) The effect of triglyceride level on PFS. (D) The effect of LDL level on PFS. PFS, progression-free survival.
